# The effects of purslane consumption on lipid profile and C‐reactive protein: A systematic review and dose–response meta‐analysis

**DOI:** 10.1002/fsn3.3555

**Published:** 2023-08-07

**Authors:** Naser Jafari, Nazgol Bahreini, Azadeh Dehghani, Yasin Lak, Seyedeh Nooshan Mirmohammadali, Simin Samavat, Amirhossein Shami, Mohammad Karimizand, Mohammad Ali Goudarzi, Omid Asbaghi

**Affiliations:** ^1^ University of Applied Science and Technology – Allameh Tabarsi Center Tehran Iran; ^2^ Student Research Committee Tabriz University of Medical Sciences Tabriz Iran; ^3^ Nutrition Research Center, School of Nutrition and Food Sciences Tabriz University of Medical Sciences Tabriz Iran; ^4^ Nutrition Research Center, Department of Community Nutrition, Faculty of Nutrition and Food Science Tabriz University of Medical Sciences Tabriz Iran; ^5^ Islamic Azad University Tehran Iran; ^6^ Department of Food, Nutrition, Dietetics and Health Kansas State University Manhattan Kansas USA; ^7^ Department of Cellular and Molecular Nutrition, School of Nutrition Sciences and Dietetics Tehran University of Medical Sciences Tehran Iran; ^8^ Student of Cellular Molecular Biology, Faculty of Science Ardabil Branch, Islamic Azad University Ardabil Iran; ^9^ Islamic Azad University, Science and Research Branch Tehran Iran; ^10^ Shahrekord Branch Islamic Azad University Shahrekord Iran; ^11^ Cancer Research Center Shahid Beheshti University of Medical sciences Tehran Iran; ^12^ Student Research Committee Shahid Beheshti University of Medical Sciences Tehran Iran

**Keywords:** C‐reactive protein, lipid profile, mechanism, meta‐analysis, purslane

## Abstract

Earlier investigations into the impact of purslane, *Portulaca oleracea*, on lipid profile and C‐reactive protein (CRP) produced contradictory findings. The effect of purslane consumption on lipid profiles and CRP was assessed in this comprehensive review and meta‐analysis. We conducted a thorough literature search in online databases, including PubMed, Scopus, the Cochrane library, and ISI Web of Science to find relevant randomized controlled trials up to June 2023. By incorporating 14 effect sizes from 13 RCTs, we were able to show that purslane consumption significantly decreases serum triglyceride (TG) (WMD: −16.72, 95% CI: −22.49, −10.96 mg/dL, *p* < .001), total cholesterol (TC) (WMD: −9.97, 95% CI: −19.86, −0.07 mg/dL, *p* = .048), and CRP (WMD: −1.22, 95% CI: −1.63, −0.80 mg/L, *p* < .001) levels in patients compared to the control group. In addition, purslane consumption significantly increases high‐density lipoprotein (HDL‐C) (WMD: 4.09, 95% CI: 1.77, 6.41 mg/dL, *p* = .001) levels. However, purslane consumption did not affect low‐density lipoprotein (LDL‐C) levels. According to a suggested optimal dosage, purslane consumption is considered to be safe up to 30 g/day. Purslane consumption can significantly improve cardiovascular health by improving lipid profile and inflammation status.

## Graphical abstract

In this review, we evaluated the effects of purslane supplementation on lipid profiles and CRP. The results of meta‐analysis showed that there was no significant impact of purslane on LDL‐C in terms of total effect size.

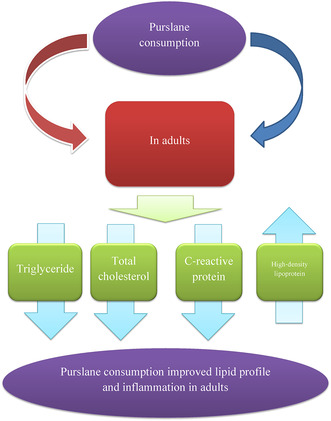

## 1 INTRODUCTION

Hyperlipidemia and chronic inflammation are major risk factors for cardiovascular disease (CVD), including coronary artery disease (CAD), cerebrovascular events, and peripheral artery disease (PAD), which is one of the leading causes of death worldwide (Benjamin et al., 2018; Nitsa et al., 2018). The occurrence of CVDs has doubled from 1990 to 2019, and over the same period, the number of deaths attributed to CVDs has risen from 12 million to a staggering 18 million (Roth et al., 2020). Globally, the expenses associated with treating CVDs are on the rise. In particular, the cost of addressing cardiovascular disorders in the United States reached a staggering 320 billion dollars in 2016 (Birger et al., 2021).

Dyslipidemia, which is characterized as increased blood total cholesterol (TC), low‐density lipoprotein cholesterol (LDL‐C), and triglycerides (TG), or decreased serum high‐density lipoprotein cholesterol (HDL‐C), significantly increases the risk of cardiovascular disease (CVD) (Hedayatnia et al., 2020; Zhao et al., 2021). There have been several efforts to sustain the lipid profile at the desired value, minimize CVD, and lower the mortality rate (Anand et al., 2018; Dong et al., 2021). It has been suggested that lipid‐lowering drugs, like HMG‐CoA reductase (HMGCR), can significantly lower the risk of morbidity and mortality attributed to CVD (Heart Protection Study Collaborative Group, 2011; Kostapanos & Elisaf, 2017). On the other hand, C‐reactive protein (CRP), an acute‐phase protein, emerged its role in vascular inflammation in CVD (Fu et al., 2020). According to recent studies, coronary artery disease, stroke, and sudden cardiac death are all closely linked to elevated CRP levels (Shrivastava et al., 2015). Therefore, reducing the CRP may decrease the incidence of CVD and may be considered as a therapeutic factor in treating CVD (Jimenez & Szalai, 2020).

Herbal supplements are cost‐effective therapeutic compounds that can affect several aspects of CVDs (Asbaghi et al., 2020; Asbaghi, Soltani, et al., 2019; Ashtary‐Larky et al., 2021; Eslampour et al., 2020). *Portulaca oleracea* or purslane is one of the herb products with numerous advantages, including anti‐diabetic, antioxidants, and anti‐hypertensive qualities (Dehghan et al., 2016). In addition, preclinical studies confirmed its effect on lipid profile (Mousa et al., 2021) and CRP (Karimi, 2018). Additionally, its application in the human population was associated with lowering the levels of some biomarkers such as LDL‐C (Gheflati et al., 2019), TG and total cholesterol (El‐Sayed, 2011), and CRP (Dehghan et al., 2016). However, some other studies had conflicting results (Esmaillzadeh et al., 2015; Zakizadeh et al., 2015). Considering the contradictory findings, we aimed at conducting a systematic review and meta‐analysis of randomized controlled trials to pool previous results and assess the impact of purslane on lipid profile and CRP levels in adults.

## 2 MATERIALS AND METHODS

This study was completed following the Preferred Reporting Items for Systematic Reviews and Meta‐Analyses (PRISMA) guideline for reporting such studies (Page et al., 2021).

### 2.1 Search strategy

Two independent researchers conducted comprehensive literature searches in PubMed, Scopus, Web of Science, and Cochrane to discover pertinent articles published before June 2023. The search was performed using the following keywords: (“Purslane” OR “Portulaca” OR “Portulaca oleracea”) AND (Intervention OR “Intervention Study” OR “Intervention Studies” OR “controlled trial” OR randomized OR randomized OR random OR randomly OR placebo OR “clinical trial” OR Trial OR “randomized controlled trial” OR “randomized clinical trial” OR RCT OR blinded OR “double‐blind” OR “double‐blinded” OR trial OR “clinical trial” OR trials OR “Pragmatic Clinical Trial” OR “Cross‐Over Studies” OR “Cross‐Over” OR “Cross‐Over Study” OR parallel OR “parallel study” OR “parallel trial”). Each database search did not include any date or language constraints. Additionally, any database search was followed by a thorough manual check of all reference lists from relevant review papers.

### 2.2 Inclusion criteria

Study eligibility was checked using the title and abstract of every identified study from the database and manual searches. The current meta‐analysis only considered studies that met the following criteria: (1) were randomized controlled trials (RCTs) or placebo‐controlled trials, (2) included adult participants >18 years old, (3) administered oral purslane alone (i.e., not in composition with other nutrients), (4) had a minimum intervention duration of 2 weeks, and (5) reported the means and standard deviations (SD) of lipid profile serum concentrations and CRP at baseline and after the study or mean change for both the intervention and control groups.

### 2.3 Excluded studies

Studies lacking a control or placebo group, interventions involving purslane consumption as part of a combination of nutrients, studies utilizing cohort, case–control, and cross‐sectional designs, animal and in vitro studies, literature reviews, and/or summary articles were all excluded from consideration.

### 2.4 Data extraction

The two researchers (OA and AD) extracted the data independently using a standardized data collection form. Information such as author's name, year, country of study, age of participants, number and gender, study design, type and dose of purslane, serum TC, TG, LDL‐C, HDL‐C, CRP concentration (before intervention and after intervention), and duration of intervention by researchers were extracted. Finally, all data units of the reported RCTs were unified to compare the average size of the effect.

### 2.5 Assessment of study quality

The Cochrane quality assessment technique was utilized in this meta‐analysis to evaluate the risk bias, random sequence generation, and allocation concealment, and the remaining four are related to performance bias, attrition bias, reporting bias, and other causes of bias. All studies were classified based on methodological defects in three domains: “high risk”, “low risk”, or “unclear risk” (Higgins et al., 2011).

### 2.6 Statistical analysis

Means and standard deviations (SDs) from stated and relevant data of RCTs were used to assess the full effect size on outcome measurements of lipid profile and CRP pre‐ to post‐purslane intervention. Weighted mean differences (WMDs) utilizing 95% confidence intervals (CIs) were represented for all variables for assessing effect sizes. The conversion of lipid profiles (TG, TC, LDL‐C, HDL‐C, and CRP levels) and CRP levels reported in mmol/L to mg/dL was done when mean changes were not recorded or when the values were provided in mmol/L but not mg/dL. Standard errors (SE), 95% confidence intervals (CIs), and interquartile ranges (IQRs) were converted to SD (Higgins et al., 2008; Hozo et al., 2005). *I* ^2^ statistic and Cochrane *Q* test and *I* ^2^ > 50% or *p*‐value <.05 were used to measure significant heterogeneity (Brondani et al., 2014). To find probabilistic sources of heterogeneity, subgroup analyses were accomplished according to the predefined criteria including trial duration (>10 vs. <10 weeks), intervention dosage (>10 vs. <10 g/day), baseline BMI (overweight/obese), baseline serum TG (<150 vs. >150 mg/dL), and TC (>200 vs. <200 mg/dL), LDL‐C (<130 vs. >130 mg/dL), HDL‐C (<40 vs. >40 mg/dL), and CRP (<3 vs. >3 mg/dL), based on median value of the included RCTs using the random‐effects models. Fractional polynomial modeling was used to assess the non‐linear potential impacts of purslane dosage (g/day) and trial duration (week) on lipid profile and CRP. Sensitivity analysis was carried out to examine the extent to which a particular study's findings might rely. To illustrate the possibility for publication bias, funnel plots and the official Egger's regression test were employed. *p*‐value <.05 was assumed as a level of significance.

## 3 RESULTS

**FIGURE 1:**
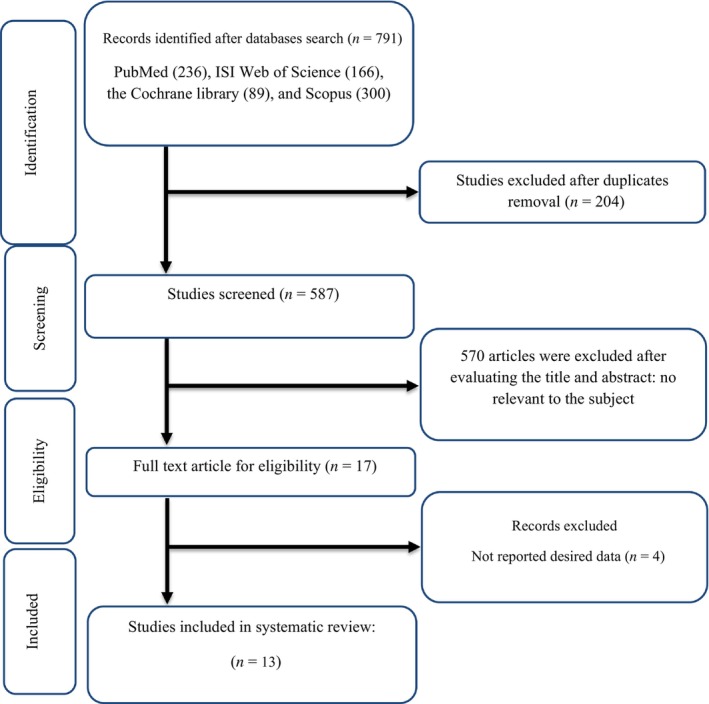
Flow chart of study selection for inclusion trials in the systematic review.

A comprehensive search of principal databases and manual searches yielded a total of 791 studies. Out of these, 204 duplicates were identified across the databases and subsequently removed. After carefully reviewing the titles and abstracts, 570 studies were excluded due to their irrelevance or failure to meet the inclusion criteria. Finally, upon assessing the full texts of the included randomized controlled trials (RCTs), four studies were excluded as they did not provide the necessary information. Finally, 13 studies (Bedakhanian, Entezari, Ghanadian, Askari, & Marsaei, 2017; Darvish Damavandi et al., 2021; Dehghan et al., 2016; Delvarianzadeh et al., 2021; El‐Sayed, 2011; Esmaillzadeh et al., 2015; Farzanegi, 2014; Gheflati et al., 2019; Ghorbanian et al., 2019; Moradi et al., 2012; Papoli et al., 2019; Parvin, Dehkordi, et al., 2013; Wainstein et al., 2016) met all inclusion criteria and were reviewed for analysis in the current systematic review and meta‐analysis (Figure 1).

### 3.1 Findings from the systematic review

Table 1 provides a comprehensive overview of the characteristics of the 13 studies and 15 effect sizes included in this systematic review. The meta‐analysis study encompassed a total of 960 adult participants, consisting of 476 cases and 484 controls, all aged above 18 years. The included randomized controlled trials (RCTs) were conducted in various settings, including Yemen (*n* = 1), Israel (*n* = 1), and Iran (*n* = 11). Each study utilized a parallel design and recommended purslane dosages ranging from 0.06 to 10 mg/day. The interventions in the included randomized controlled trials (RCTs) lasted for a duration of 4–16 weeks and were conducted on populations with distinct characteristics. Specifically, eight studies focused on oral purslane consumption in patients with type 2 diabetes, one study targeted hypercholesterolemia patients, one study involved schizophrenic patients, two studies involved patients with metabolic syndrome, and two studies examined individuals diagnosed with non‐alcoholic fatty liver disease. The mean age of the participants ranged from 40 ± 17.52 to 61.17 ± 4.88 years. Among the 13 selected RCTs with a parallel design, only one study had a crossover design. The Cochrane risk of bias assessment tool was used to evaluate the studies, revealing that some studies exhibited a minimal risk of bias in most of the assessed domains (Table 2).

**TABLE 1:** Characteristic of included studies in meta‐analysis.

Study	Country	Study design	Sex	Trial duration (week)	Participants	Means age (year)		Means BMI (kg/m^2^)		Intervention			Sample size	
						IG	CG	IG	CG	Treatment group	Intervention dose (g/day)	Control	IG	CG
El‐Sayed (2011)	Yemen	Paralell, R, PC, DB	M/F (F:10, M:20)	8	Type 2 diabetes	40 ± 17.52	40 ± 17.52	31.03 ± 3.8	32.27 ± 5.2	Purslane	10	Metformin	15	15
Moradi et al. (2012)	Iran	Paralell, R	M/F: 93	8	Hypercholesterolemia patients	44 ± 9.6	49 ± 11.6	27 ± 3.9	26 ± 4.9	Purslane	50	Lovastatin	41	52
Parvin, Farzane‐Dehkordi, et al. (2013)	Iran	Paralell, R, PC, DB	M/F: (F:19, M:41)	8	Schizophrenic patients	43.76 ± 10.96	45.26 ± 10.03	NR	NR	Purslane	1	Control diet	30	30
Farzanegi (2014)	Iran	Paralell, R, PC	F: 14	8	Type 2 diabetes	51.17 ± 4.88	50.83 ± 6.79	29.88 ± 4.34	30.71 ± 4.34	Purslane & exercise	7.5	Control diet & execise	7	7
Farzanegi (2014)	Iran	Paralell, R, PC	F: 14	8	Type 2 diabetes	52.33 ± 4.08	50.17 ± 5.34	29.01 ± 4.34	29.37 ± 4.55	Purslane	7.5	Control diet	7	7
Esmaillzadeh et al. (2015)	Iran	Crossover, R, PC	M/F: 48	5	Type 2 diabetes	51.4 ± 6.09	51.4 ± 6.09	28.99 ± 3.9	28.8 ± 3.9	purslane	10	Control diet	48	48
Dehghan et al., 2016	Iran	Paralell, R, PC, DB	F: 98	16	Type 2 diabetes	52.33 ± 4.08	50.17 ± 5.34	29 ± 5	29.9 ± 7.3	Purslane	7.5	Placebo	49	49
Dehghan et al., 2016	Iran	Paralell, R, PC, DB	F: 98	16	Type 2 diabetes	61.17 ± 4.88	58.83 ± 6.79	29.8 ± 6.4	29.5 ± 7.2	Purslane & aerobic training	7.5	Placebo & aerobic training	49	49
Wainstein et al. (2016)	Israel	Paralell, R, PC, DB	M/F (F:22, M:41)	12	Type 2 diabetes	52.4 ± 7.9	58.3 ± 10.8	29.9 ± 3.8	29.1 ± 3.6	Purslane	0.18	Placebo	31	32
Bedakhanian, Entezari, Ghanadian, Askari, and Maracy (2017)	Iran	Paralell, R, PC	M: 78	8	Metabolic syndrome	46.5 ± 7.6	47.8 ± 6.5	28.38 ± 1.79	28.57 ± 2.15	Purslane	0.06	Control diet	39	39
Gheflati et al. (2019)	Iran	Paralell, R, PC	M/F (F:48, M:12)	8	Non‐alcoholic fatty liver disease	40.07 ± 9.52	39.81 ± 8.84	32.77 ± 3.63	31.09 ± 3.24	Purslane	10	Control diet	27	27
Ghorbanian et al. (2019)	Iran	Paralell, R, PC	F: 20	8	Non‐active girls	20–30	20–30	27 ± 2.6	28.21 ± 9.8	Purslane	1.2	Control diet	10	10
Papoli et al. (2019)	Iran	Paralell, R, PC	F: 64	12	Metabolic syndrome	42.16 ± 5.48	43.16 ± 8.33	28.23 ± 4.43	26.3 ± 3.72	Purslane	10	Control diet	32	32
Darvish Damavandi et al. (2021)	Iran	Paralell, R, PC, DB	M/F (F:31, M:43)	12	Non‐alcoholic fatty liver disease	46.18 ± 9.71	46.05 ± 10.09	31.56 ± 3.78	31.83 ± 3.97	Purslane	0.3	Placebo	37	37
Delvarianzadeh et al. (2021)	Iran	Paralell, R, PC, DB	M/F (F:51, M:53)	4	Type 2 diabetes	53.5 ± 6.75	53.6 ± 6.34	NR	NR	Purslane	10	Control diet	54	50

Abbreviations: CG, control group; DB: double‐blind; F, Female; IG, intervention group; M, Male; NR, not reported.

**TABLE 2:** Quality assessment.

Study	Random sequence generation	Allocation concealment	Selective reporting	Other sources of bias	Blinding (participants and personnel)	Blinding (outcome assessment)	Incomplete outcome data	General quality
El‐Sayed (2011)	L	L	L	L	L	U	L	L
Moradi et al. (2012)	L	H	L	H	H	H	L	H
Parvin, Farzane‐Dehkordi, et al. (2013)	L	H	L	H	L	U	L	M
Farzanegi (2014)	L	H	L	H	H	H	L	H
Esmaillzadeh et al. (2015)	L	L	L	L	H	H	L	M
Dehghan et al., 2016	L	H	L	H	L	U	L	M
Wainstein et al. (2016)	L	L	L	L	L	U	L	L
Bedakhanian, Entezari, Ghanadian, Askari, and Marsaei (2017)	L	L	L	H	H	H	L	H
Gheflati et al. (2019)	L	L	L	L	H	H	L	M
Ghorbanian et al. (2019)	L	H	L	H	H	H	L	H
Papoli et al. (2019)	L	L	L	L	H	H	L	M
Darvish Damavandi et al. (2021)	L	L	L	L	L	U	L	L
Delvarianzadeh et al. (2021)	L	H	L	H	L	U	L	M

Abbreviations: H, high‐risk of bias; L, low‐risk of bias; U, unclear‐risk of bias.

### 3.2 Findings from the meta‐analysis

#### 3.2.1 The effect of purslane consumption on serum TG concentrations

**FIGURE 2:**
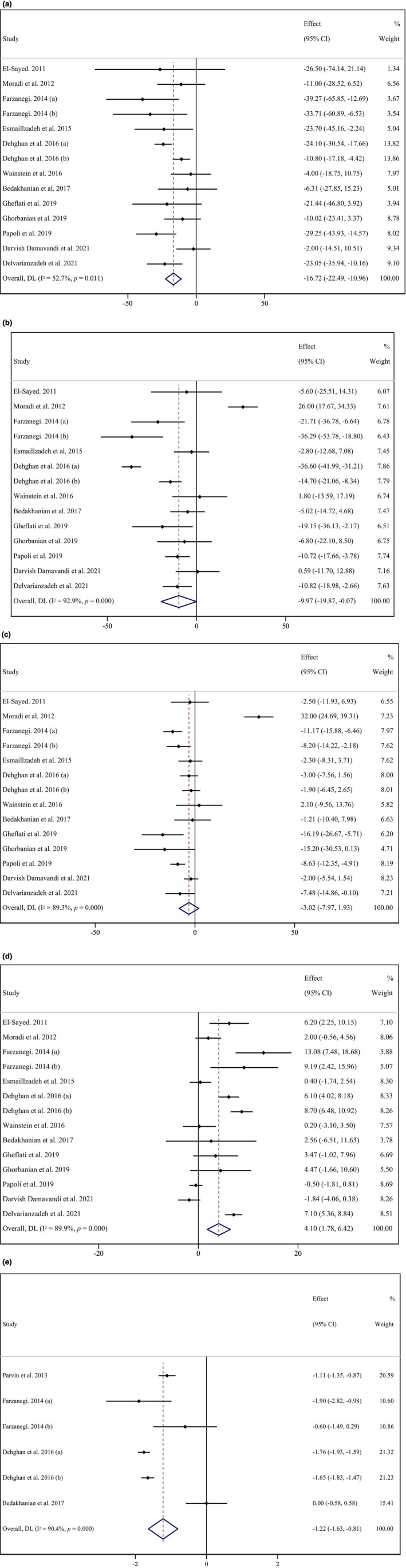
Forest plot detailing weighted mean difference and 95% confidence intervals (CIs) for the effects of purslane consumption on (a) TG (mg/dL); (b) TC (mg/dL); (c) LDL (mg/dL); (d) HDL (mg/dL); (e) and CRP (mg/dL).

Our analysis revealed a significant reduction in serum triglyceride (TG) levels in patients who consumed purslane compared to the control group (weighted mean difference [WMD]: −16.72, 95% CI: −22.49, −10.96 mg/dL, *p* < .001), combining data from 14 effect sizes extracted from 13 RCTs (see Figure 2a). However, there was significant heterogeneity among the studies (*I* ^2^: 52.7, *p* = .01). To explore potential sources of heterogeneity, we conducted subgroup analyses based on several factors, including sex, trial duration (≤8 vs. >8 weeks), intervention dosage (≥10 vs. <10 g/day), baseline BMI (overweight 25–29.9 vs. obese ≥30 kg/m^2^), health status (diabetes vs. non‐diabetes), and baseline TG levels (<150 vs. ≥150 mg/dL), using the median values across the included RCTs as cutoffs. The subgroup analyses revealed that purslane consumption significantly reduced serum TG concentrations in studies involving overweight subjects, particularly in females with high TG levels (refer to Table 3). These findings provide insights into the potential impact of purslane consumption on specific subgroups and highlight the influence of certain participant characteristics on the observed heterogeneity.

**TABLE 3:** Subgroup analyses of purslane supplementation on lipid profile and C‐reactive protein.

	Effect size	WMD (95% CI)	*p*‐Value	Heterogeneity	
				*p* heterogeneity	*I* ^2^
Subgroup analyses of purslane on TG level (mg/dL)					
Overall effect	14	−16.72 (−22.48, −10.96)	**<.001**	.011	52.7%
Sex					
Both sexes	8	−12.17 (−19.27, −5.07)	**.001**	.274	19.7%
Female	6	−21.00 (−29.71, −12.29)	**<.001**	.008	68.0%
Trial duration (week)					
≤8	9	−18.45 (−24.91, −11.98)	**<.001**	.445	0.0%
>8	5	−14.31 (−23.90, −4.73)	**.003**	.001	79.1%
Intervention dose (g/day)					
≥10	6	−22.49 (−29.93, −15.06)	**<.001**	.774	0.0%
<10	8	−13.99 (−21.94, −6.05)	**.001**	.003	67.9%
Baseline BMI (kg/m^2^)					
Overweight (25–29.9)	10	−17.38 (−24.09, −10.67)	**<.001**	.013	57.0%
Obese (≥30)	3	−9.08 (−23.44, 5.26)	.215	.286	20.1%
Health status					
Diabetes	8	−19.64 (−27.31, −11.97)	**<.001**	.021	57.6%
Non‐diabetes	6	−12.70 (−21.58, −3.82)	**.005**	.124	42.2%
Baseline serum TG (mg/dL)					
<150	1	−10.02 (−23.41, 3.37)	.143	–	–
>150	13	−17.41 (−23.60, −11.22)	**<.001**	.009	54.7%
Subgroup analyses of purslane on TC level (mg/dL)					
Overall effect	14	−9.97 (−19.86, −0.07)	**.048**	<.001	92.9%
Sex					
Both sexes	8	−1.33 (−12.16, 9.48)	.809	<.001	86.2%
Female	6	−20.98 (−32.50, −9.46)	**<.001**	<.001	89.9%
Trial duration (week)					
≤8	9	−8.38 (−20.53, 3.77)	.177	<.001	89.2%
>8	5	−12.75 (−27.01, 1.50)	.080	<.001	93.8%
Intervention dose (gr/day)					
≥10	6	−3.35 (−17.35, 10.64)	.639	<.001	91.4%
<10	8	−14.95 (−26.60, −3.29)	**.012**	<.001	90.4%
Baseline BMI (kg/m^2^)					
Overweight (25–29.9)	10	−10.49 (−23.36, 2.36)	.110	<.001	94.9%
Obese (≥30)	3	−7.09 (−19.19, 5.00)	.251	.182	41.3%
Health status					
Diabetes	8	−16.07 (−27.03, −5.12)	**.004**	<.001	89.8%
Non‐diabetes	6	−1.98 (−16.01, 12.04)	.781	<.001	90.6%
Baseline serum TC (mg/dL)					
<200	11	−14.25 (−22.87, −5.63)	**.001**	<.001	88.2%
>200	3	5.83 (−17.81, 29.49)	.628	<.001	92.2%
Subgroup analyses of purslane on LDL‐C level (mg/dL)					
Overall effect	14	−3.02 (−7.97, 1.93)	.232	<.001	89.3%
Sex					
Both sexes	8	0.44 (−8.61, 9.51)	.923	<.001	92.1%
Female	6	−6.91 (−10.35, −3.48)	**<.001**	.026	60.7%
Trial duration (week)					
≤8	9	−3.29 (−12.63, 6.05)	.490	<.001	92.9%
>8	5	−3.59 (−6.80, −0.38)	**.028**	.056	56.7%
Intervention dose (gr/day)					
≥10	6	−0.75 (−13.21, 11.70)	.906	<.001	95.2%
<10	8	−4.66 (−7.90, −1.42)	.005	.022	57.1%
Baseline BMI (kg/m^2^)					
Overweight (25–29.9)	10	−1.60 (−8.34, 5.13)	.641	<.001	92.1%
Obese (≥30)	3	−5.88 (−13.76, 2.00)	.144	.042	68.5%
Health status					
Diabetes	8	−4.91 (−7.88, −1.94)	.001	.070	46.6%
Non‐diabetes	6	−1.35 (−13.09, 10.39)	**.821**	<.001	95.2%
Baseline serum LDL‐C (mg/dL)					
<130	7	−5.01 (−8.21, −1.82)	**.002**	.019	60.3%
>130	7	−0.20 (−12.18, 11.77)	.973	<.001	94.3%
Subgroup analyses of purslane on HDL‐C level (mg/dL)					
Overall effect	14	4.09 (1.77, 6.41)	**.001**	<.001	89.9%
Sex					
Both sexes	8	2.44 (−0.31, 5.21)	.083	<.001	86.2%
Female	6	6.54 (2.05, 11.04)	**.004**	<.001	93.5%
Trial duration (week)					
≤8	9	5.10 (2.43, 7.77)	**<.001**	<.001	79.1%
>8	5	2.54 (−1.51, 6.59)	.219	<.001	94.8%
Intervention dose (gr/day)					
≥10	6	2.99 (−0.05, 6.04)	.054	<.001	90.7%
<10	8	5.14 (1.38, 8.90)	**.007**	<.001	89.2%
Baseline BMI (kg/m^2^)					
Overweight (25–29.9)	10	4.28 (1.47, 7.08)	**.003**	<.001	89.4%
Obese (≥30)	3	2.39 (−2.92, 7.72)	.378	.001	85.7%
Health status					
Diabetes	8	5.95 (3.26, 8.65)	**<.001**	<.001	86.4%
Non‐diabetes	6	0.61 (−1.17, 2.40)	.498	.070	50.9%
Baseline serum HDL‐C (mg/dL)					
<40	7	6.70 (4.07, 9.32)	**<.001**	.001	73.2%
>40	7	1.72 (−1.15, 4.60)	.241	<.001	90.1%
Subgroup analyses of purslane on CRP level (mg/L)					
Overall effect	6	−1.22 (−1.63, −0.80)	**<.001**	<.001	90.4%
Sex					
Female	2	−0.58 (−1.67, 0.49)	.289	.001	91.7%
Both sexes	4	−1.64 (−1.88, −1.40)	**<.001**	.080	55.6%
Trial duration (week)					
≤8	4	−0.87 (−1.57, −0.17)	**.015**	.001	82.0%
>8	2	−1.70 (−1.83, −1.58)	**<.001**	.380	0.0%
Health status					
Diabetes	4	−1.64 (−1.88, −1.40)	**<.001**	.080	55.6%
Non‐diabetes	2	−0.58 (−1.67, 0.49)	.289	.001	91.7%
Baseline serum CRP (mg/L)					
<3	1	−1.11 (−1.34, −0.87)	**<.001**	–	–
≥3	5	−1.24 (−1.72, −0.76)	**<.001**	<.001	89.5%

*Note*: Bold values are statistically significant *p* < .005.

Abbreviations: BMI, body mass index; CI, confidence interval; CRP, c‐reactive protein; HDL‐C, High‐density lipoprotein; LDL‐C, Low‐density lipoprotein; TC, total cholesterols; TG, triglycerides; WMD, weighted mean differences.

#### 3.2.2 The effect of purslane consumption on serum TC concentrations

Upon analyzing the data from 14 effects extracted from 13 studies, a significant difference in blood concentrations of total cholesterol (TC) was observed between the intervention group and the control group following purslane consumption (weighted mean difference [WMD]: −9.97 mg/dL, 95% CI: −19.86 to −0.07, *p* = .048) (see Figure 2b). However, substantial study heterogeneity was detected (*I* ^2^ = 92.9%, *p* < .001). Further subgroup analyses revealed that purslane consumption had a significant impact on serum TC concentrations in diabetic patients (WMD: −16.07 mg/dL, 95% CI: −27.03 to −5.12, *p* = .004), females (WMD: −20.98 mg/dL, 95% CI: −32.50 to −9.46, *p* < .001), when the intervention dose was <10 mg/day (WMD: −14.95 mg/dL, 95% CI: −26.60 to −3.29, *p* = .012), and when the baseline serum TC was below 200 mg/dL (WMD: −14.25 mg/dL, 95% CI: −22.87 to −5.63, *p* = .001) (refer to Table 3). These findings highlight the potential benefits of purslane consumption in specific subgroups, particularly in diabetic patients, females, and individuals with lower baseline TC levels.

#### 3.2.3 The effect of purslane consumption on serum LDL‐C concentrations

In this meta‐analysis, we considered 14 effect sizes extracted from 13 trials. When comparing the interventional group to the control group, a quantitative analysis indicated that purslane consumption did not have a significant effect on LDL cholesterol (LDL‐C) values (weighted mean difference [WMD]: −3.02 mg/dL, 95% CI: −7.97 to 1.93, *p* = .232). The overall heterogeneity among the studies was found to be significantly high (*I* ^2^ = 89.3%, *p* < .001) (refer to Figure 2c). However, subgroup analyses revealed that among females, purslane consumption had a significant impact on serum LDL‐C concentrations (WMD: −6.91 mg/dL, 95% CI: −10.35 to −3.48, *p* < .001). Furthermore, when the study duration was >8 weeks (WMD: −3.59 mg/dL, 95% CI: −6.80 to −0.38, *p* = .028), in diabetic patients (WMD: −4.91 mg/dL, 95% CI: −7.88 to −1.94, *p* = .001), and in individuals with a baseline serum LDL‐C level below 130 mg/dL (WMD: −5.01 mg/dL, 95% CI: −8.21 to −1.82, *p* = .002), purslane consumption demonstrated a significant effect (refer to Table 3). These findings suggest that the impact of purslane consumption on LDL‐C levels may vary depending on specific subgroups, such as gender, study duration, diabetic status, and baseline LDL‐C levels.

#### 3.2.4 The effect of purslane consumption on serum HDL‐C concentrations

A total of 14 effect sizes were analyzed to examine the effects of purslane consumption on serum HDL cholesterol (HDL‐C) concentrations. The quantitative meta‐analysis revealed a significant mean effect of purslane consumption on serum HDL‐C concentrations in the intervention group compared to the control group (weighted mean difference [WMD]: 4.09 mg/dL, 95% CI: 1.77–6.41, *p* < .001). However, notable between‐study heterogeneity was observed (*I* ^2^: 89.9%, *p* < .001) (refer to Figure 2d). Further subgroup analyses were conducted based on the predefined categories. The results demonstrated a significant increase in HDL‐C levels with purslane consumption (WMD: 4.09 mg/dL, 95% CI: 1.77–6.41, *p* < .001). Subgroup analysis revealed significant results when the study duration was <8 weeks (WMD: 5.10 mg/dL, 95% CI: 2.43–7.77, *p* < .001), the intervention dose was <10 g/day (WMD: 5.14 mg/dL, 95% CI: 1.38–8.90, *p* = .007), the intervention was performed on overweight participants (WMD: 4.28 mg/dL, 95% CI: 1.47–8.65, *p* < .001), and the baseline serum HDL‐C was below 40 mg/dL (WMD: 6.70, 95% CI: 4.07–9.32, *p* < .001) (refer to Table 3). These findings suggest that purslane consumption has a significant positive impact on HDL‐C levels, particularly in shorter‐duration studies, with lower intervention doses, in overweight participants, and in individuals with lower baseline HDL‐C levels.

#### 3.2.5 The effect of purslane consumption on serum CRP concentrations

Upon comparing different intervention strategies between participants and control groups, the analysis showed a significant reduction in blood concentrations of CRP with purslane consumption (weighted mean difference [WMD]: −1.22 mg/L, 95% CI: −1.63 to −0.80, *p* < .001) based on six effect sizes from trials. There was also significant between‐study heterogeneity observed overall (*I* ^2^: 90.4%, *p* < .001) (refer to Figure 2e). Subgroup analyses were conducted based on participants with diabetes, baseline blood CRP levels (≥3 mg/L vs. <3 mg/L), and trial duration (≥8 weeks vs. <8 weeks). The findings indicated that only when both sexes were analyzed, purslane consumption had a significant effect on serum CRP concentrations (WMD: −1.64 mg/L, 95% CI: −1.88 to −1.40, *p* < .001). Similarly, when the intervention was performed on individuals with diabetes, a significant effect on CRP levels was observed (WMD: −1.64 mg/dL, 95% CI: −1.88 to −1.40, *p* < .001) (refer to Table 3). These results suggest that purslane consumption has a significant impact on reducing CRP levels, particularly when considering both sexes and in individuals with diabetes.

#### 3.2.6 Publication bias and sensitivity analyses

**FIGURE 3:**
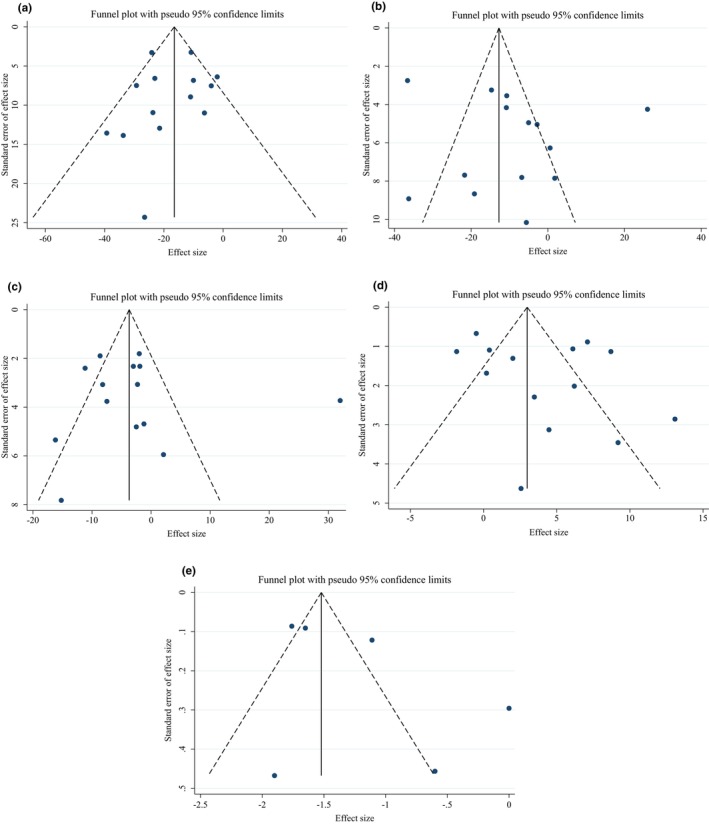
Funnel plots for the effect of purslane consumption on (a) TG (mg/dL); (b) TC (mg/dL); (c) LDL (mg/dL); (d) HDL (mg/dL); (e) and CRP (mg/dL).

We observed no evidence of publication bias for TG (*p* = .668), TC (*p* = .342), LDL‐C (*p* = .362), HDL‐C (*p* = .287), and CRP (*p* = .218) based on visual inspection of funnel plots and Egger's regression test (Figure 3a–e). In conclusion, the results of the sensitivity analysis revealed that there was no noticeable impact of any particular study on the overall effect sizes of TG serum concentrations, LDL‐C, HDL‐C, and CRP. However, about TC after removing studies by (El‐Sayed, 2011) (WMD: −10.25 mg/dL, 95% CI: −20.57, 0.06), (Farzanegi, 2014) (WMD: −9.11 mg/dL, 95% CI: −19.52, 1.28), (Farzanegi, 2014) (WMD: −8.16 mg/dL, 95% CI: −18.34, 2.02), (Esmaillzadeh et al., 2015) (WMD: −10.55 mg/dL, 95% CI: −21.12, 0.02), (Dehghan et al., 2016) (WMD: −7.46 mg/dL, 95% CI: −15.53, 0.60), (Dehghan et al., 2016) (WMD: −9.59 mg/dL, 95% CI: −20.81, 1.61), (Bedakhanian, Entezari, Ghanadian, Askari, & Maracy, 2017; Bedakhanian, Entezari, Ghanadian, Askari, & Marsaei, 2017) (WMD: −10.37 mg/dL, 95% CI: −21.01, 0.25), (Gheflati et al., 2019) (WMD: −9.33 mg/dL, 95% CI: −19.70, 1.04), (Ghorbanian et al., 2019) (WMD: −10.20 mg/dL, 95% CI: −20.62, 0.21), (Papoli et al., 2019) (WMD: −9.92 mg/dL, 95% CI: −21.00, 1.14), (Delvarianzadeh et al., 2021) (WMD: −9.91 mg/dL, 95% CI: −20.78, 0.95), overall effect was significantly changed.

#### 3.2.7 None‐linear and linear dose–response analyses

**FIGURE 4:**
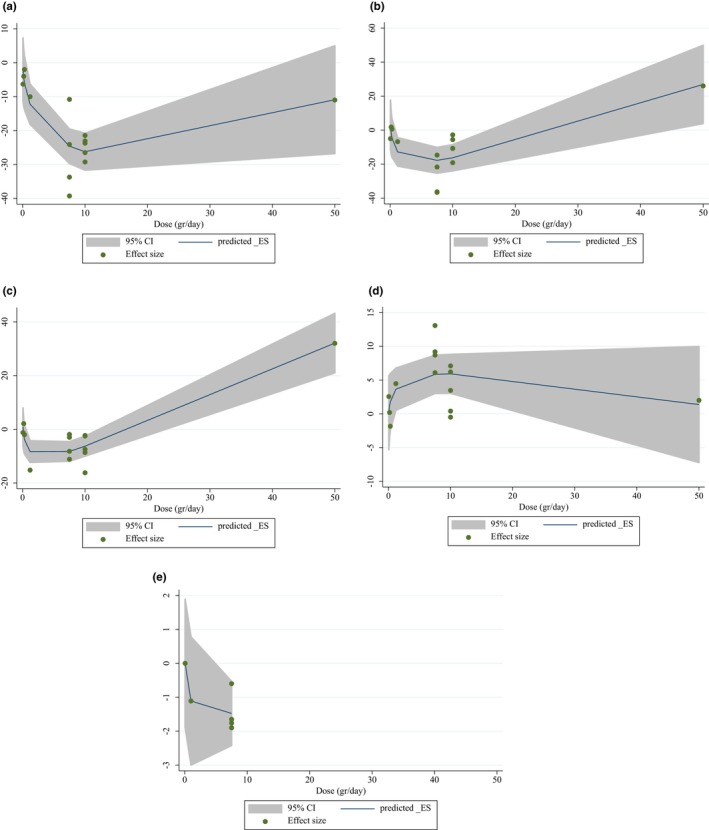
Non‐linear dose–response relations between purslane consumption and absolute mean differences. Dose–response relations between dose (gr/day) and absolute mean differences in (a) TG (mg/dL); (b) TC (mg/dL); (c) LDL (mg/dL); (d) HDL (mg/dL); (e) and CRP (mg/dL).

**FIGURE 5:**
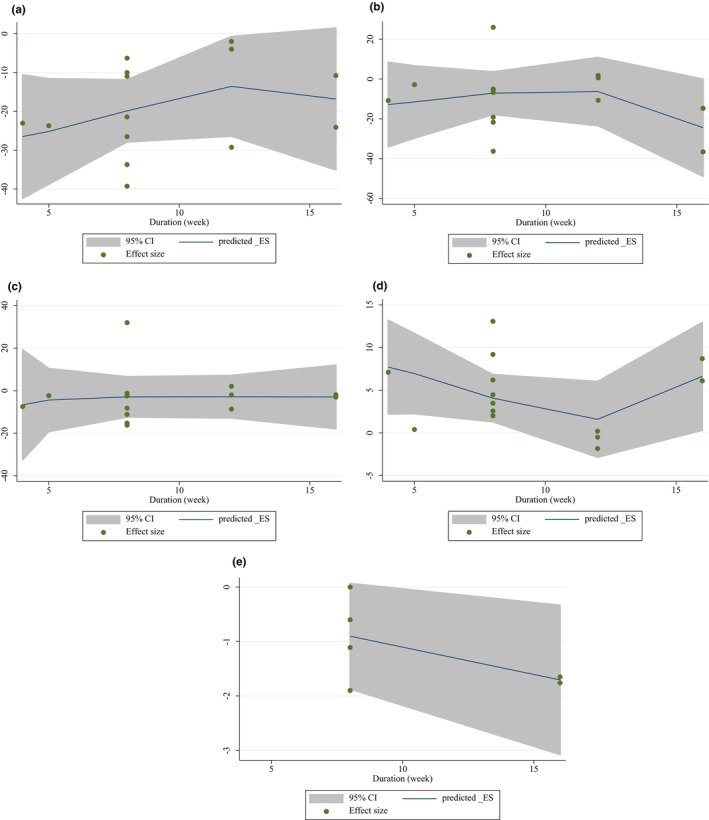
Non‐linear dose–response relations between purslane consumption and absolute mean differences. Dose–response relations between duration of intervention (week) and absolute mean differences in (a) TG (mg/dL); (b) TC (mg/dL); (c) LDL (mg/dL); (d) HDL (mg/dL); (e) and CRP (mg/dL).

**FIGURE 6:**
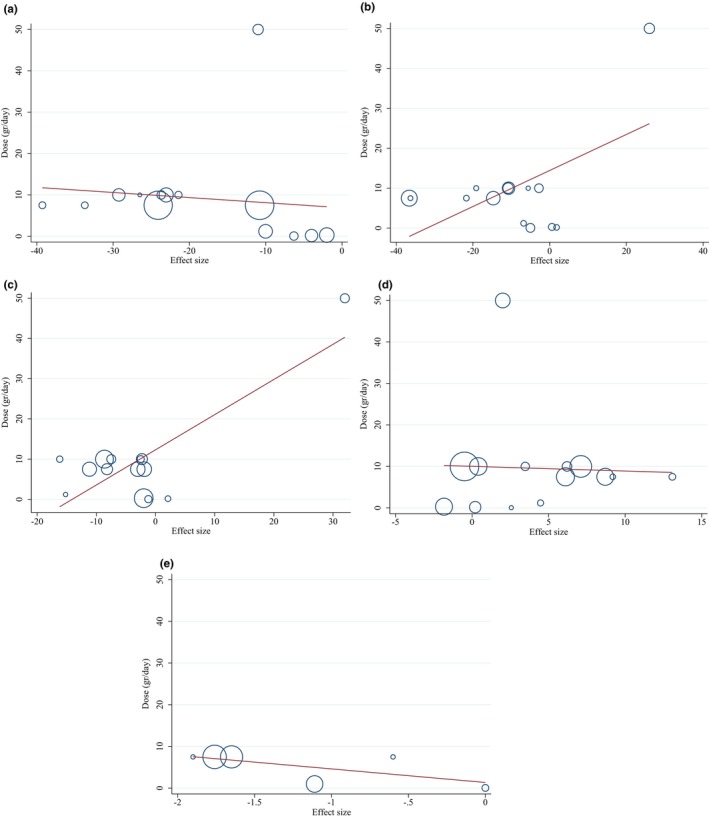
Linear dose–response relations between purslane consumption and absolute mean differences. Dose–response relations between dose (gr/day) and absolute mean differences in (a) TG (mg/dL); (b) TC (mg/dL); (c) LDL (mg/dL); (d) HDL (mg/dL); (e) and CRP (mg/dL).

**FIGURE 7:**
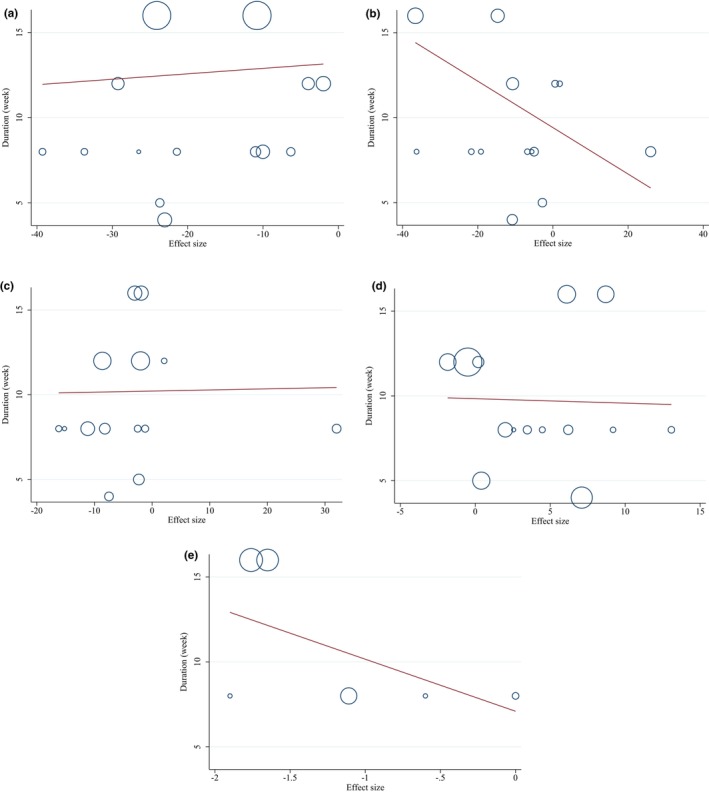
Linear dose–response relations between purslane consumption and absolute mean differences. Dose–response relations between duration of intervention (week) and absolute mean differences in (a) TG (mg/dL); (b) TC (mg/dL); (c) LDL (mg/dL); (d) HDL (mg/dL); (e) and CRP (mg/dL).

The non‐linear analysis revealed a significant relationship between the dose of intervention and changes in triglycerides (TG) (*p* = .001), total cholesterol (TC) (*p* = .016), and low‐density lipoprotein cholesterol (LDL‐C) (*p* = .017) (refer to Figure 4a–e). However, no non‐linear relationship was found between the duration of intervention and changes in any of the variables (refer to Figure 5a–e). Furthermore, a linear relationship was observed between intervention dose and changes in TC (*p* = .041) and LDL‐C (*p* = .001) (refer to Figure 6a–e). On the other hand, no association was found between the study duration and changes in any of the variables (refer to Figure 7a–e). These findings suggest that the dose of intervention may play a role in influencing changes in TG, TC, and LDL‐C levels, while the duration of intervention does not appear to have a significant impact on these variables.

#### 3.2.8 Grading of evidence

The GRADE protocol was used to recognize the certainty of the evidence (Table 4). The effect evaluation of inconsistency for all factors was downgraded with low quality for severe heterogeneity. Results showed serious limitations in the imprecision of LDL. The body of the systematic review and meta‐analysis of evidence was rated as moderate overall quality.

**TABLE 4:** GRADE profile The effects of purslane supplementation on lipid profile and C‐reactive protein.

Quality assessment						Summary of findings		Quality of evidence
Outcomes	Risk of bias	Inconsistency	Indirectness	Imprecision	Publication bias	Number of intervention/control	WMD (95% CI)	
TG	No serious limitations	Serious limitations^a^	No serious limitations	No serious limitations	No serious limitations	900 (446/454)	−16.72 (−22.48, −10.96)	⊕⊕⊕◯ Moderate
TC	No serious limitations	Very serious limitations^a^	No serious limitations	No serious limitations	No serious limitations	900 (446/454)	−9.97 (−19.86, −0.07)	⊕⊕◯◯ Low
LDL	No serious limitations	Very serious limitations^a^	No serious limitations	Serious limitations^b^	No serious limitations	900 (446/454)	−3.02 (−7.97, 1.93)	⊕◯◯◯ Very low
HDL	No serious limitations	Very serious limitations^a^	No serious limitations	No serious limitations	No serious limitations	900 (446/454)	4.09 (1.77, 6.41)	⊕⊕◯◯ Low
CRP	No serious limitations	Very serious limitations^a^	No serious limitations	No serious limitations	No serious limitations	362 (181/181)	−1.22 (−1.63, −0.80)	⊕⊕◯◯ Low

^a^ The test for heterogeneity is significant for TG (*I* ^2^: 52.7%), TC (*I* ^2^: 92.9%), LDL (*I* ^2^: 89.3%), HDL (*I* ^2^: 89.9%), and CRP (*I* ^2^: 90.4%).

^b^ The effect of purslane on LDL was insignificant.

## 4 DISCUSSION

In this systematic review and meta‐analysis, we analyzed data from thirteen RCTs to assess the effects of purslane consumption on lipid profile and CRP levels in adults. The main findings of our study indicate that compared to the control group, purslane supplementation led to significant reductions in TC, TG, and CRP levels, while increasing HDL‐C levels. However, we did not observe a significant effect of purslane on LDL‐C levels. These findings suggest that purslane consumption can have beneficial effects on lipid profile and inflammation markers, which may contribute to improved cardiovascular health.

The results of this meta‐analysis demonstrate that purslane supplementation significantly reduced levels of TG, TC, and CRP. These findings are consistent with previous systematic reviews of clinical trials, which have also shown that purslane supplementation may effectively reduce these markers in various populations, including overweight individuals and those with diabetes (Acedo et al., 2012; El‐Sayed, 2011; Gheflati et al., 2019; Parvin, Dehkordi, et al., 2013; Varlamov et al., 2015). However, it is important to note that in a specific clinical trial involving obese adults, purslane supplementation did not lead to a significant change in TG levels when compared to a placebo (Gheflati et al., 2019). It should be noted that short‐term study duration has been considered as a limitation in these studies. Therefore, further longitudinal studies are needed to shed light on this issue.

In this study, we found no significant effect of purslane supplementation on LDL‐C. Several previous studies also could not find a significant effect of purslane on LDL‐C (Zakizadeh et al., 2015; بداخانيان, انتظاري, قناديان, عسکري,,, & مراثي, 2017). For example, one study involving patients with type 2 diabetes found that consuming bulk bread containing 10% Portulaca powder for 4 weeks did not result in any significant changes in LDL‐C levels. However, another clinical trial focusing on patients with non‐alcoholic fatty liver disease showed a significant reduction in LDL‐C after 8 weeks of purslane supplementation compared to a placebo (Gheflati et al., 2019). Although our meta‐analysis did not show a significant effect of purslane on LDL‐C overall, subgroup analyses revealed significant effects in studies conducted on subjects with diabetes and those with intervention durations exceeding 8 weeks. This suggests that longer intervention periods may be necessary to observe a meaningful impact on LDL‐C levels. However, due to the limited number of available studies on the effects of purslane supplementation on LDL‐C, further clinical trials are needed to provide more conclusive evidence in this area.

In contrast, our meta‐regression analysis revealed that purslane supplementation had a positive impact on HDL levels in adults who were overweight, had diabetes, or had a baseline serum HDL‐C level below 40. However, we did not observe any significant change in patients who were obese or when the supplementation was administered for a longer duration (Papoli et al., 2019).

The exact biological mechanism underlying the effects of purslane has not yet been fully elucidated. However, purslane is known to be rich in various nutrients, and it is an excellent source of the antioxidant, vitamins, L‐ norepinephrine, and glutathione in purslane also may play a role in the observed hypocholestrolemic effects Several studies have actually demonstrated that the consumption of purslane can lead to a reduction in oxidized LDL‐cholesterol (OxLDL) levels. It is believed that the antioxidant compounds present in purslane play a crucial role in preventing the formation of lipoperoxides during the process of LDL oxidation. On the other hand, high concentrations of Melatonin, a free radical scavenger, are recently identified in purslane. Melatonin also reduces TC in rats with high cholesterol diet (Simopoulos et al., 1992). Another active substance in purslane seed is beta‐sitosterol which is a phytosterol. Studies have found that beta‐sitosterol has cholesterol (Ostlund Jr, 2007) and LDL (Abumweis et al., 2008) lowering effects, increases the expression of VEGF (vascular endothelial growth factor) while modulating inflammation and regulation the immune systems. The effectiveness of purslane seed consumption is in lowering cholesterol levels explained by the synergistic effect of both phytosterols and unsaturated fatty acids (Micallef & Garg, 2008). Purslane is truly a valuable source of omega‐3 fatty acids, which have been found to have beneficial effects on triglyceride (TG) levels. Research has indicated that omega‐3 polyunsaturated fatty acids (PUFAs) can lower TG levels. This effect is believed to be due to the strong affinity of omega‐3 fatty acids for the peroxisome proliferator‐activated receptor (PPAR), resulting in an enhancement of beta oxidation and fatty acid metabolism. Moreover, omega‐3 PUFAs may also contribute to reducing the synthesis of TG in the liver by inhibiting the activity of acyl‐CoA1,2‐diacylglycerol acyltransferase (Chauhan et al., 2017; Hoy & Keating, 2009). Some evidence detected that HDL often is likely to increase when there was a marked reduction in serum TG concentrations (Skulas‐Ray et al., 2008). Omega‐3 PUFAs inhibits SREBP‐1c and lipogenesis, while increases PPAR‐α and fatty acid oxidation (Spadaro et al., 2008). Through these mechanisms, PPAR‐α agonists might be able to lower TG and increase HDL levels (Balachandran, 2019). Furthermore, according to some animal studies purslane polysaccharides might be responsible for significant increased concentration of HDL‐C (Kumar et al.). Hyperlipidemia enhance production of proinflammatory markers such as CRP, reactive oxygen species (ROS) and reduce anti‐inflammatory cytokine and adiponectin (Green et al., 2011). One of the most clinically significant inflammatory indicators for predicting the prevalence of cardiovascular illnesses is CRP (Boncler et al., 2019; Califf, 2018) which is even a stronger biomarker than low‐density lipoprotein cholesterol (Asbaghi, Fouladvand, et al., 2019). In the study of (Nehring & Patel, 2017) purslane consumption reduced total CRP levels by 0.33 mg/dL (95% CI: 0.66, 0.004, *p* = .047). The observed inhibitory effects of unsaturated fatty acids on NF‐κB in patients could potentially explain the mechanism behind the benefits of omega‐3 fatty acids. Some studies have reported the inhibitory effect of unsaturated fatty acids, including omega‐3, on CRP levels in diabetic patients, although the changes in CRP levels may not have been statistically significant. Nevertheless, the significant reduction of CRP in other studies suggests that there may be a synergistic effect of various compounds present in purslane seeds, working together to provide these beneficial outcomes. It is crucial to note that just four research totaled in our analysis for CRP levels (Bedakhanian, Entezari, Ghanadian, Askari, & Maracy, 2017; Dehghan et al., 2016; Farzanegi, 2014; Parvin, Dehkordi, et al., 2013) with significant heterogeneity detected (*I* ^2^: 90.4%, *p* < .001). The various study designs, lengths of interventions, and participant numbers may account for the contradictory outcomes on biomarkers of inflammation.

Purslane is a well‐tolerated natural remedy that is frequently employed in herbal remedies as a safe supplementary treatment (El‐Sayed, 2011). The studies included in the current meta‐analysis did not identify any significant negative impacts of purslane use. A recommended optimal dosage of purslane eating indicates that up to 30 g/day of purslane consumption has no negative effects (Reid, 1987). The only issue is the high amounts of oxalic acid, which should be ingested with caution due to its potential to increase kidney stone risk, particularly in at‐risk persons (Palaniswamy et al., 2004). Additionally, oxalate acid may impair the digestion of some minerals, including calcium and iron (Bataille & Fournier, 2001). However, just like many herbal medicines, it should be used with caution in some situations, like pregnancy (Izzo et al., 2016). In assessing the overall findings, it is important to take into account the limitations of the current study. Firstly, the number of studies included in the meta‐analysis was relatively small, which could potentially limit the generalizability of the results. Additionally, the outcomes of the included studies may have been influenced by a lack of adjustment for potential confounding factors during the analysis, which could introduce bias and affect the accuracy of the findings. Numerous research studies have examined the relationships between lipid profiles and physical activity (Durstine et al., 2001), diet (Obarzanek et al., 2001), and smoking (Kar et al., 2016). Unfortunately, the majority of the included studies did not evaluate these characteristics. Therefore, the overall findings may be confounded by these parameters.

## AUTHOR CONTRIBUTIONS

**Naser Jafari:** Writing – original draft (equal). **Nazgol Bahreini:** Writing – original draft (equal). **Azadeh Dehghani:** Writing – review and editing (equal). **Yasin Lak:** Writing – original draft (equal). **Amirhossein Shami:** Writing – original draft (equal).

## CONFLICT OF INTEREST STATEMENT

The authors declared that there is no conflict of interest.

## Data Availability

Data available on request from the authors.
